# Phase Ib/II study to evaluate the safety and antitumor activity of durvalumab (MEDI4736) and tremelimumab as monotherapy or in combination, in patients with recurrent or metastatic gastric/gastroesophageal junction adenocarcinoma

**DOI:** 10.1186/2051-1426-3-S2-P157

**Published:** 2015-11-04

**Authors:** Ronan J Kelly, Ki Chung, Yu Gu, Keith E Steele, Marlon C Rebelatto, Paul B Robbins, Fatemeh Tavakkoli, Joyson J Karakunnel, Dominic W Lai, Khaldoun Almhanna

**Affiliations:** 1 The Sidney Kimmel Comprehensive Cancer Center of Johns Hopkins, Baltimore, MD, USA; 2 GHS Cancer Institute, Spartanburg, SC, USA; 3 MedImmune, Gaithersburg, MD, USA; 4 Moffitt Cancer Center, Tampa, FL, USA

## Background

Despite improvements in diagnosis, surgical techniques, and multidisciplinary approaches to patient care, the median survival of patients with metastatic gastroesophageal adenocarcinoma is less than one year. Chemotherapy is the mainstay of treatment in patients with metastatic disease, but is associated with significant toxicity. There is still, therefore, a significant unmet clinical need in this patient population. Blockade of immune checkpoints is one of the most promising novel approaches in cancer treatment. Blocking programmed cell death ligand-1 (PD-L1) or cytotoxic T-lymphocyte-associated antigen-4 (CTLA-4) immune checkpoints with anti-PD-L1 or anti-CTLA-4 antibodies has shown clinical promise in other solid tumors. Durvalumab (MEDI4736) is a selective, high affinity human IgG1 monoclonal antibody (mAb) that blocks PD-L1 binding to programmed cell death-1 (PD-1) (IC_50_ 0.1 nM) and CD80 (IC_50_ 0.04 nM). Tremelimumab is a selective human IgG2 mAb inhibitor of CTLA-4, which promotes T-cell activity through CTLA-4 inhibition. Preclinical data suggest that combining immunotherapies may result in superior antitumor activity versus monotherapy, leading to higher and more durable response rates and improved overall and progression-free survival (PFS).

## Methods

This is a randomized, multicenter, open-label Phase Ib/II study to investigate the safety and antitumor activity of durvalumab and tremelimumab, given as monotherapy or in combination, in patients with histologically- or cytologically-confirmed recurrent or metastatic gastric or gastroesophageal junction adenocarcinoma who have progressed on, or are refractory to standard regimens (NCT02340975). The Phase Ib part of the study will identify the doses and schedules to be used in Phase II. In Phase II, patients will be enrolled in one of four treatment arms. The objectives for Phase Ib are to assess the safety and tolerability (primary objective) and the antitumor activity (objective response rate [ORR], and PFS at six months [PFS-6]; secondary objectives) of durvalumab in combination with tremelimumab. The primary objectives for Phase II are to assess the antitumor activity (ORR and PFS-6) of the monotherapies and combination therapy; the secondary objectives are to further assess safety, tolerability, and antitumor activity (disease control rate, duration of response, overall survival), and to investigate correlations between PD-L1 expression and clinical activity. Exploratory objectives include evaluation of pharmacokinetics, pharmacodynamics, and immunogenicity. Recruitment is ongoing, with an estimated enrollment of 174 patients.

## Trial registration

ClinicalTrials.gov identifier NCT02340975.

